# Bio-Inspired Voronoi-Based Porous Tubular Structure Design and Crashworthiness Properties

**DOI:** 10.3390/ma19050997

**Published:** 2026-03-05

**Authors:** Mengfei Han, Qinxi Dong, Hui Wang

**Affiliations:** 1 School of Civil Engineering and Architecture, Hainan University, Haikou 570228, China; mengfeihan@hainanu.edu.cn; 2 Department of Civil Engineering, Shandong Jiaotong University, Jinan 250357, China; 903008@sdjtu.edu.cn

**Keywords:** biomimetic design, Voronoi structure, cellular structure, crashworthiness, energy absorption

## Abstract

Porous tubular structures are of significant interest in engineering due to their exceptional potential for lightweight design, energy absorption, and multifunctional integration. Inspired by the unique net architecture of natural luffa sponges, this study introduces a novel design approach for such structure, namely bio-inspired Voronoi Tube (BVT). This design employs Voronoi tessellation patterns, parametrically controlled through the spatial distribution of seed points and integrates iterative optimization algorithms, to achieve precise coordinated regulation over the randomness and continuity of the resulting spatial network, closely mimicking the biological paradigm. Then, specimens are fabricated via additive manufacturing and then quasi-statically compressed axially, followed by systematic mechanical testing of the base material. The experimental results are analyzed to reveal the BVT structure’s mechanical responses and simultaneously validate finite-element simulation model. Subsequently, a systematic numerical analysis is performed to further understand the deformation mechanisms of the BVT structure and the influence of key geometric parameters. The results indicate that the iteratively optimized BVT structure successfully replicates the characteristic energy absorption behavior of the natural luffa sponge, confirming the effectiveness of the bio-inspired design. A rise in diameter from 0.6 mm to 1.0 mm results in a 78.32% increase in the specific energy absorption (SEA). Under identical mass conditions, tailored adjustments to the geometry can enhance the SEA by up to 34.57%.

## 1. Introduction

Cellular solid materials, as a class of porous materials characterized by open porosity, have attracted extensive attention in cutting-edge fields such as aerospace, automotive engineering, optoelectronics, and biomedical engineering, with their interdisciplinary research value and application potential becoming increasingly prominent [1,2,3]. Cellular materials can be constructed through random or ordered arrangements of unit cells, among which non-random cellular structures are characterized by the periodic distribution of primitive units, forming highly ordered cellular topologies [4,5,6]. Macroscopically, cellular structures exhibit superior comprehensive properties compared to traditional materials, particularly outstanding performance in mechanical aspects, including low density, high specific stiffness, and efficient energy absorption capacity [7,8]. By optimizing internal geometries, engineers can produce components with exceptional robustness and light weight, which renders them ideal for practical applications. Based on the geometric characteristics of spatial distribution of unit cells, cellular structures can be mainly classified into three categories: honeycomb structures [9], foam structures [10], and rod-based structures [11,12]. Among these, three-dimensional (3D) rod-based structures, distinguished by their clear structural order, provide an ideal platform for directional design and active control of mechanical properties, thereby emerging as a significant direction in both current research and applied development.

In the design methodology of cellular materials, three major categories can be summarized: triply periodic minimal surface structures based on mathematical geometric models, topology-optimized structures based on numerical calculations, and bio-inspired designs inspired by natural biological structures [13]. As a representative design paradigm, bio-inspired design focuses on extracting optimized structural features from biological systems—shaped by long-term evolution—and translating them into engineered cellular structures with specific geometric topologies based on biomimetic principles. Examples include the porous configurations in deep-sea glass sponge structure [14], the elytra of the ironclad beetles [15], bamboo and honeycomb structural characteristics [16]. According to differences in morphological generation methods and construction principles, existing bio-inspired cellular structures can be further divided into curved bio-inspired cellular structures [15,17,18,19], straight-rod bio-inspired cellular structures [20,21,22,23], hollow-section bio-inspired cellular structures [16], and boundary-coincident bio-inspired cellular structures [14,24,25].

**Figure 1:**
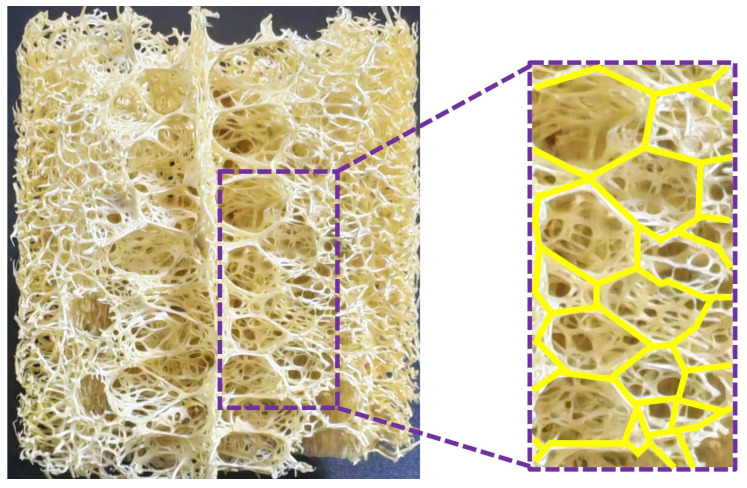
Longitudinal section imaging of luffa sponge structure.

The irregularity of cellular structures has a significant influence on their mechanical behavior. Cellular structures with irregular geometries can even surpass closed-cell foam materials in compressive performance [26]. It is noteworthy that conventional cellular structures typically exhibit anisotropic energy absorption characteristics, whereas the application of an irregularization treatment can enhance both their isotropic response and energy absorption efficiency [27,28]. Against this background, the distance-metric-based Voronoi diagram, as a spatial partitioning method, demonstrates unique potential [29,30]. This configuration appears in 3D space as a non-periodic network composed of polyhedral cells, which exhibit statistical variations in face count, edge count, and vertex connectivity, forming a continuously interconnected pore system through shared geometric elements. This geometric irregularity endows the Voronoi model with high design flexibility, making it valuable in fields such as hollow-structure modeling, mechanical property optimization, and lightweight design [31]. Various mechanical configurations have been developed based on Voronoi diagrams, which can be categorized according to spatial morphology into skeleton-cubic types [32,33], foam-like types [34,35,36], and composite-filled types [37,38,39]. These structures demonstrate exceptional mechanical performance, characterized by their distance-based partitioning system that inherently generates an efficient space-filling pattern. This design strategy not only integrates lightweight and high-strength properties but also optimizes material distribution to minimize redundancy, thereby significantly enhancing overall stiffness and deformation resistance [40]. Furthermore, the continuity of connections between the polygonal units contributes to improved structural stability, facilitating uniform load distribution and mitigating localized stress concentration under applied forces. In 3D space, the Voronoi diagram manifests as a non-periodic network structure composed of polyhedral unit cells, whose topological characteristics closely resemble the microscopic morphology of natural porous luffa sponge (as shown in Figure 1).

This study introduces a novel bio-inspired structure, the Bio-Inspired Voronoi Tubular (BVT) structure, by constructing a 3D Voronoi-based spatial network that emulates the architecture of natural luffa sponge. Unlike conventional cellular structures with regular periodic arrays, the proposed approach generates a continuously distributed, stochastic-yet-controllable porous geometry. By parametrically regulating seed point density and integrating iterative optimization, the method enables coordinated control over structural randomness, continuity, and density. The BVT structures are fabricated via additive manufacturing and experimentally validated through finite element simulations, followed by a systematic parametric analysis of their geometric parameters. This modeling strategy not only accurately imitates the porous morphology of natural luffa sponge but also enables tunable mechanical properties through geometric parameter adjustment, offering a novel pathway for optimizing energy-absorbing structures.

## 2. Materials and Methods

This section presents the detailed materials and methods used in this work. It is structured into four subsections to systematically outline the methodology.

### 2.1. Bio-Inspired Structural Design

**Figure 2:**
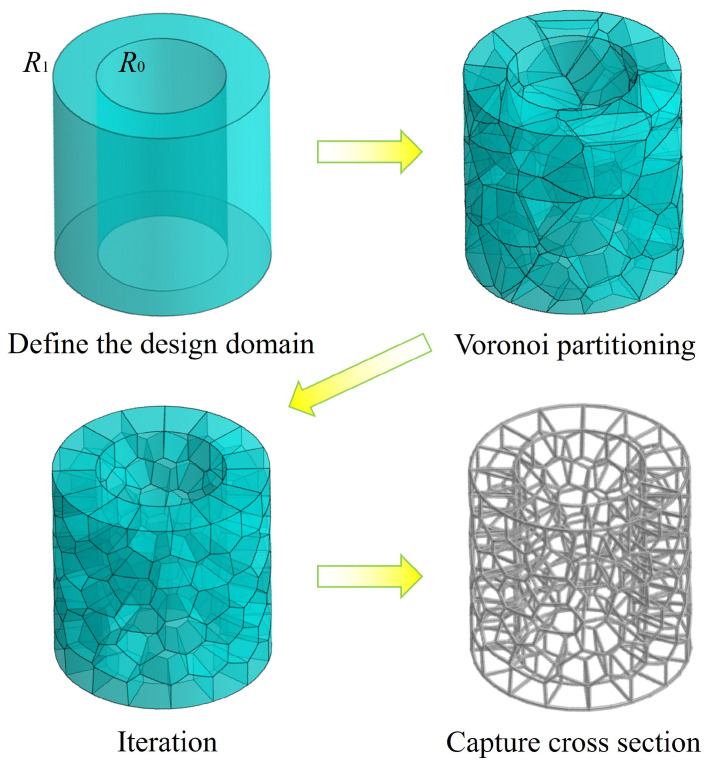
Schematic diagram of the BVT structure design process based on 3D Voronoi diagrams.

As illustrated in Figure 2, a cylindrical geometric model is established with defined dimensional parameters: inner radius *R*_0_, outer radius *R*_1_, outer diameter *D*_1_, and height *H*. Following this, seed points are generated within the design domain of the cylindrical volume. Based on their spatial distribution, Voronoi polyhedral partition is constructed within the modeling domain. During the initial network generation phase, the number of Voronoi seed points (*N*) is controlled to regulate the network density. This value of *N* also corresponds to the number of Voronoi cells, thereby influencing the final density of the network structure.

In the modeling process, the skeleton lines constructed from the Voronoi polyhedral unit’s boundaries generated by segmentation are prone to significant geometric interference when forming an irregular network structure, which is specifically manifested as unintended spatial intersections of multiple structural members. Notably, this leads to a pronounced stress concentration effect in the intersecting regions of the members. To address this issue, an iterative optimization strategy is employed in this study. First, multiple rounds of optimization calculations are performed on the initially partitioned spatial polyhedral units. By dynamically adjusting the positions of seed points to progressively achieve a state of ideal distribution, resulting in spatially well-distributed Voronoi polyhedral units.

Here we briefly introduce the Voronoi principle. Three-dimensional Voronoi tessellation is a mathematical method for discretizing a given spatial structure, whose core principle is spatial nearest-neighbor partitioning. Given a set of predefined seed points, the plane is divided into several non-overlapping convex polygonal regions, such that any point within each region is closer to its corresponding seed point than to any other seed point. Its mathematical definition is as follows.

Given a set of *n* seed points in an *m*-dimensional Euclidean space, the perpendicular bisecting hyperplane of any two points *p_i_* and *p_j_* divides the space into two half-spaces. Define *H*(*p_i_*, *p_j_*) as the half-space containing *p_i_*, i.e., the set of all points closer to *p_i_*:

(1) $$H(p_{i},p_{j})=\{x\in R^{m}\left|d(x,p_{i})\leq \right.d(x,p_{j})\}$$

where *d*(*x*, *p*) denotes the Euclidean distance between two points. Then the *m*-dimensional Voronoi cell *V*(*p_i_*) corresponding to the seed point *p_i_* can be expressed as the intersection of all such half-spaces:

(2) $$V(p_{i})=\overset{n}{\underset{\begin{array}{c} j=1\\ j\neq i \end{array}}{\cap }}H(p_{i},p_{j})$$

The complete m-dimensional Voronoi diagram can be written as:

(3) $$\gamma (p)=\{V(p_{i}),\ldots ,V(p_{n})\}$$

Following the optimization of the spatial polyhedral units, boundary curves are extracted and a uniform cross-sectional dimension (diameter *d*) is applied to form a consistent rod-based framework. This completes the construction of a BVT structure. This modeling approach not only preserves the characteristics of the Voronoi network but also effectively improves the uniformity of the network distribution, thereby establishing a superior geometric foundation for subsequent mechanical performance analysis.

### 2.2. Compression Experiments

#### 2.2.1. Material Properties of the Base Material

**Figure 3:**
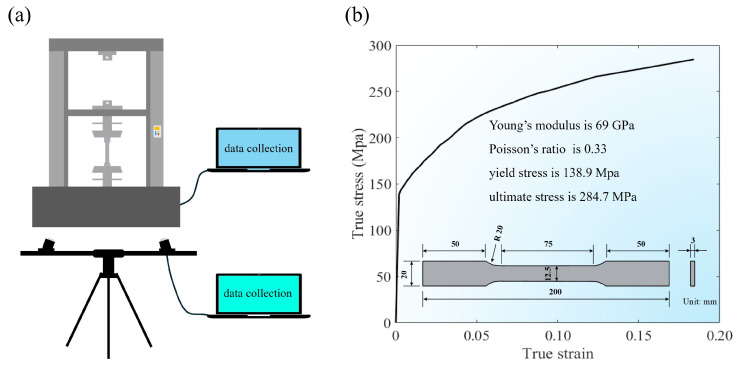
Material properties: (**a**) tensile test of specimens and (**b**) dimensions of specimen with engineering stress–strain curves of AA6061.

In the investigation of cellular structures, aluminum alloy serves as one primary material due to its advantageous combination of low density and manufacturability. This study characterizes aluminum alloy AA6061 fundamental mechanical properties through standardized tensile testing in accordance with ASTM E8E8M-22 [41]. Tensile specimens are fabricated using selective laser sintering (SLS) additive manufacturing, maintaining a dimensional tolerance of ±0.10 mm. The fabricated specimens exhibit a consistent average density of 2700 kg/m^3^. Quasi-static tensile loading is applied via a servo-electric universal testing machine (model ETM105D, WANCE Ltd., Shenzhen, China) with 100 kN load. Full-field strain measurement is accomplished via a non-contact approach using three-dimensional digital image correlation, with the experimental setup depicted in Figure 3a. Utilizing this optical method allows for precise quantification of deformation. The mechanical response of AA6061 is illustrated in Figure 3b. It is represented by the averaged engineering stress–strain curve obtained from repeated tests.

#### 2.2.2. Quasi-Static Compression Tests for the Present BVT Structures

**Figure 4:**
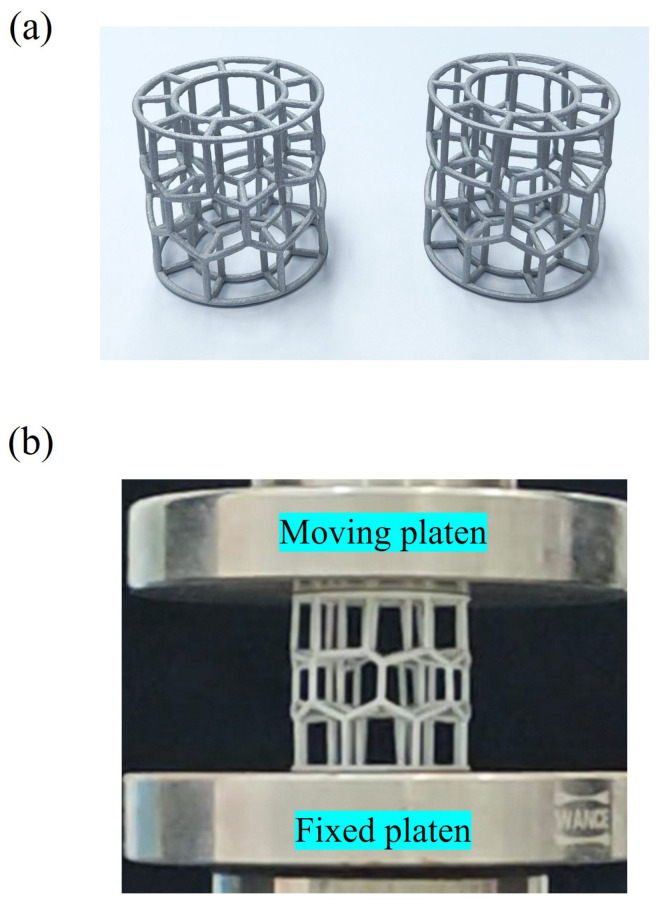
Schematic diagram of the test setup: (**a**) specimen and (**b**) compression test.

The two specimens employed for compression testing are presented in Figure 4, which fabricated from aluminum alloy AA6061. Their geometric dimensions are defined as follows: a wall thickness of *d* = 2 mm, *R*_0_ = 15 mm, *R*_1_ = 25 mm, and *H* = 50 mm. Considering the current process constraints of additive manufacturing, a conservative meshing density (*N* = 25) is adopted to minimize potential forming defects associated with high-density fine cellular structures. The measured masses of the two specimens are 18.64 g and 18.35 g, respectively, as determined by a precision balance. The resulting mass deviation of less than 2% indicates a high level of manufacturing repeatability.

Quasi-static axial compression testing is conducted on a universal testing machine (model ETM105D, WANCE Ltd., Shenzhen, China). A displacement-controlled loading regime is applied at a constant rate of 2.00 mm/min. Prior to each test, the compression platen is leveled and the specimen is concentrically aligned on the stationary base to ensure axial loading. Throughout compression, the full load–displacement response is continuously recorded by a data acquisition system, from which key mechanical parameters are subsequently derived.

### 2.3. Crashworthiness Indicators

To assess the energy absorption capacity and crashworthiness of the BVT, the following five indicators are employed [42]: energy absorption (*EA*), specific energy absorption (*SEA*), initial peak force (*IPF*), crushing force efficiency (*CFE*), and the undulation of load-carrying (*ULC*).

Firstly, it is necessary to determine the effective compression distance *d*. This is achieved by introducing the energy absorption efficiency η, defined by the relationship:

(4) $$\eta (\sigma_{d})=\frac{\int_{0}^{\varepsilon_{d}}\sigma (\varepsilon )d\varepsilon }{\sigma_{d}}$$

where $\varepsilon_{d}$ represents the densification strain, and $\sigma_{d}$ represents the axial stress at the corresponding position. Subsequently, *d* can be solved by solving the following equations:

(5) $$\frac{d\eta (\varepsilon_{d})}{d\varepsilon }=0$$

(6) $$d=H\cdot \varepsilon_{d}$$

*EA* quantifies the total energy dissipated by the structure under impact loading, which is mathematically expressed as the integral of the instantaneous crushing force over the axial crushing distance:

(7) $$EA=\int_{0}^{d}F(x)dx$$

where *F*(*x*) denotes the instantaneous crushing force.

*SEA* denotes as the energy absorbed per unit mass of the structure, which can be formulated as:

(8) $$SEA=\frac{EA}{m}$$

where *m* corresponds to the total mass of structure.

The Mean Crushing Force (*MCF*) characterizes the structure’s average load-bearing capacity throughout the deformation process and is calculated as:

(9) $$MCF=\frac{EA}{d}$$

*CFE* is introduced to assess the stability and uniformity of the force response during crushing, defined by the ratio:

(10) $$CFE=\frac{MCF}{IPF}\times 100\%$$

where the *IPF* represents the maximum force recorded prior to the onset of densification, typically corresponding to the first prominent peak on the force–displacement curve.

*ULC* is defined to evaluate the crushing force efficiency:

(11) $$ULC=\frac{\int_{0}^{d}\left|F(x)-MCF\right|dx}{\int_{0}^{d}F(x)dx}$$

### 2.4. Finite Element Simulations

#### 2.4.1. Finite Element Model

**Figure 5:**
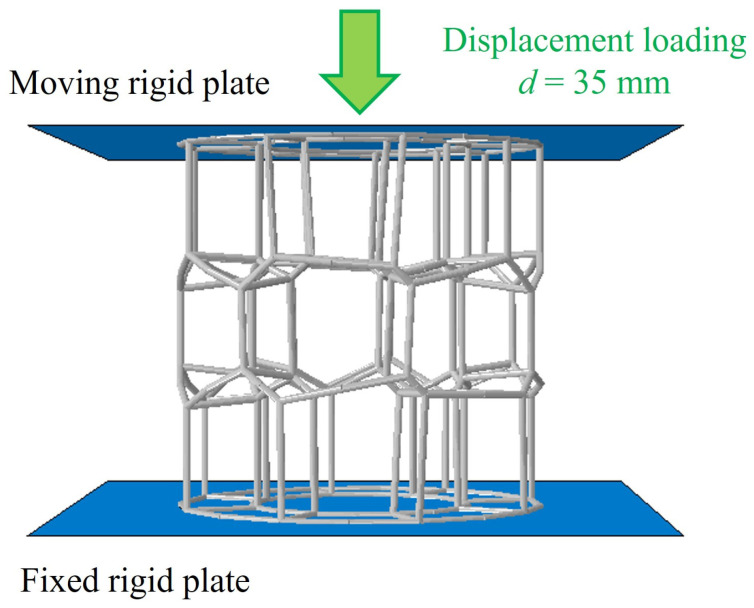
A schematic diagram of the finite element model.

A finite element model is developed to simulate the compression process of the BVT, as illustrated in Figure 5. The model comprises three main components: a moving rigid plate, a stationary supporting rigid plate, and the BVT structure. Both the upper and lower plates are modeled as analytical rigid bodies. The upper plate is assigned a constant downward velocity of 30 mm/s, while the other is constrained. The lower supporting plate is fully fixed in all degrees of freedom. General contact is applied throughout the model to account for interaction effects, utilizing a friction coefficient of 0.2 [43,44] in the tangential direction of contacting surfaces and a hard-contact condition in the normal direction. Moreover, beam element is employed for numerical simulation owing to their high computational efficiency and convenience in parametric design and iterative optimization, which is accepted in the preliminary design and optimization of porous structures. Through systematic convergence analysis, an optimal element size of 0.7 mm is determined. To ensure consistency between the simulation and experimental conditions, the loading displacement is strictly set at 35 mm in accordance with the testing standard.

#### 2.4.2. Model Verification and Deformation Mode Analysis

**Figure 6:**
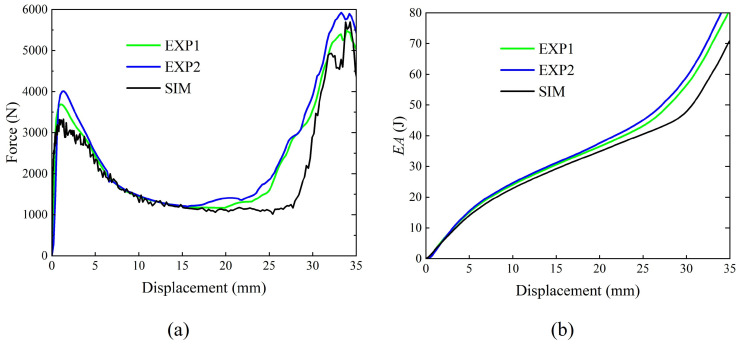
Comparison of finite element results and test results: (**a**) force–displacement curves, (**b**) energy absorption curves.

The comparative analysis between simulation and experimental results (Figure 6) reveals that the experimentally measured force–displacement curve enters the densification stage earlier than its simulated counterpart. This discrepancy is primarily attributed to the reduced sensitivity of the beam-element modeling approach to cross-sectional dimensions, which in turn delays the simulated structural contact response relative to the experimental test. However, the energy absorption curve in Figure 6b exhibits only minor deviations between the simulation and experiment, indicating the high computational accuracy of the finite element model.

**Figure 7:**
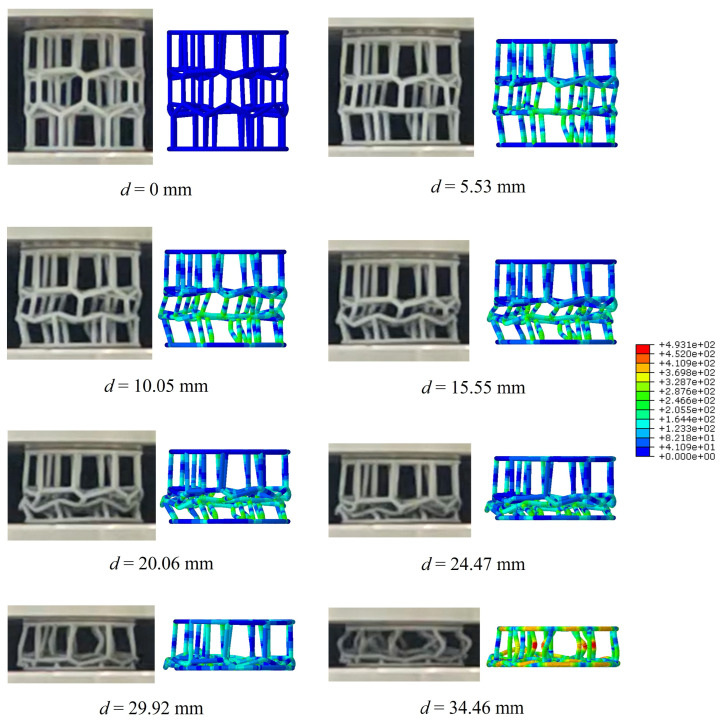
Deformation mode of BVT under axial quasi-static compression.

Furthermore, as illustrated in Figure 7, the simulation accurately captures the three-stage progressive buckling behavior observed experimentally in the BVT structure under quasi-static compression. The process involves buckling initiation in the intermediate layer, propagation from one end toward the opposite side, and eventually full densification. This deformation pattern can be explained by the following mechanisms. Firstly, owing to the limited density of the network, the intermediate layer contains a higher concentration of relatively short rod structures, which under axial compression reach their buckling critical load earlier. Then, the intrinsic geometric non-uniformity of the Voronoi network leads to buckling in the central region, which mechanically induces instability in the adjacent network layer on one side. Finally, this mechanical disturbance propagates along the axial direction, resulting in progressive buckling that develops outward from the middle layer toward both ends. In terms of force–displacement behavior, the load-carrying capacity increases upon buckling initiation at one end and remains elevated until buckling occurs at the far end. This demonstrates a sustained load-sharing interaction among the different network layers, which collectively enhances the overall bearing capacity.

To systematically investigate the influence of network density on energy-absorption performance, the subsequent study continues to employ the beam-element-based finite element method, which maintains simulation accuracy while preserving computational efficiency through an optimized higher-density Voronoi network.

## 3. Comparative Analysis of Optimization Iterations

**Figure 8:**
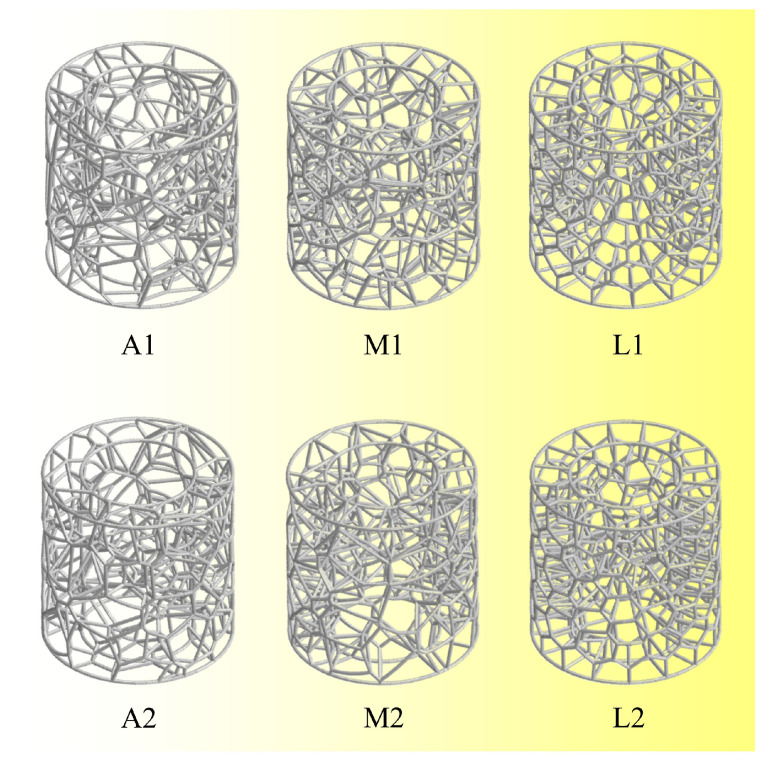
Schematic diagram of BVT structure with different numbers of optimization iterations.

To examine the effect of optimization iteration times on the energy absorption performance of BVT structures, this section compares the energy absorption capacities of structures generated with different numbers of iterations. As illustrated in Figure 8, three groups of BVT structures are constructed with the same geometric parameters (*d* = 1 mm, *N* = 125, *R*_0_ = 15 mm, *R*_1_ = 25 mm, *H* = 50 mm) but varying iteration times. Each group contains two samples for comparative analysis: the base group (A1, A2) retains the initial network without iteration; the moderate-iteration group (M1, M2) undergoes 10 optimization iterations; and the high-iteration group (L1, L2) undergoes 50 optimization iterations. With increasing iteration count, the network distribution of the BVT structures progressively becomes more uniform. Finite element simulations are then conducted to analyze the mechanical responses of all structures under axial compression.

**Figure 9:**
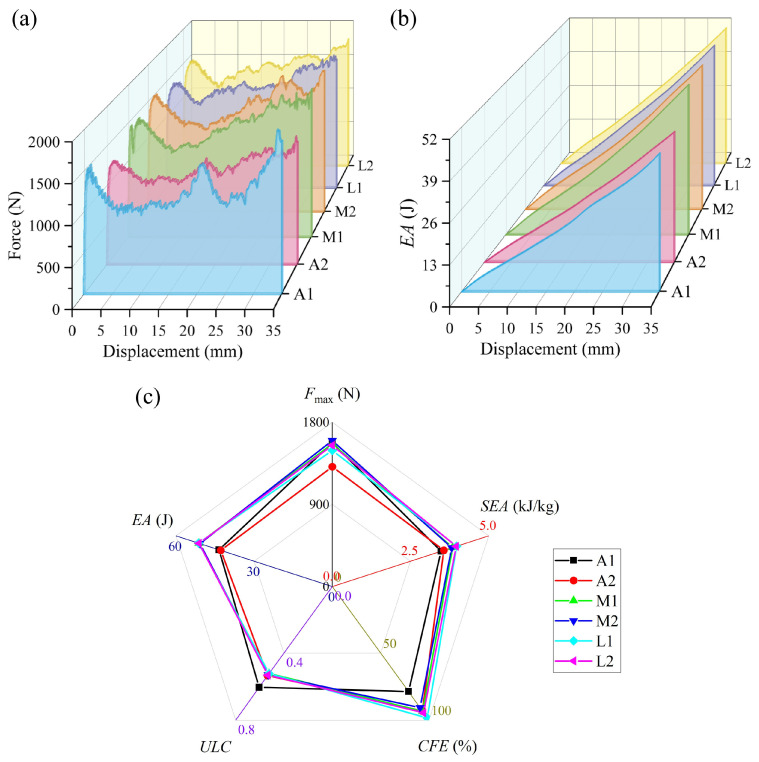
Comparison of energy absorption performance of BVT under different optimization iteration times: (**a**) force–displacement curves, (**b**) energy absorption curves, (**c**) energy absorption performance indicators.

Figure 9 shows the simulated comparison of the energy absorption performance of BVT structures under different iteration times. As shown in Figure 9a, the force–displacement curves exhibit distinct differences across iteration levels: the low-iteration structure A1 displays pronounced fluctuations with two clear peaks, reflecting its local buckling and non-synergistic failure characteristics. In contrast, the moderate- and high-iteration structures demonstrate ideal compressive responses typical of efficient energy absorption—characterized by a smooth, stable plateau region with minimal fluctuations, indicating that orderly progressive deformation enables uniform stress distribution and stable energy dissipation. With increasing iteration times, the energy absorption performance of the BVT structures improves notably. Specifically, the *SEA* shows a clear upward trend: the high-iteration sample L1 achieves an 8.50% higher *SEA* compared with the moderate-iteration sample M2, and a 36.21% increase relative to the base sample A1. Overall, the average *SEA* of the high-iteration group is 23.66% higher than that of the base group and 6.36% higher than that of the moderate-iteration group. It is noteworthy that the base structures without iteration exhibit considerable variability in energy absorption performance (with differences of 19.95% in *SEA* and 14.27% in *CFE*), whereas the optimized moderate- and high-iteration structures demonstrate much more consistent energy absorption characteristics (*CFE* variations are controlled within 5%). Meanwhile, the average *CFE* values for the base, moderate-iteration, and high-iteration groups are 86.01%, 92.11%, and 96.40%, respectively. Furthermore, the *ULC* of the iteratively optimized BVT structures is generally lower than that of the base structures, indicating a smoother energy absorption process, which aligns with the design requirement of low fluctuation for energy-absorbing structures. In summary, the optimization iteration count significantly enhances the energy absorption performance of BVT structures, while the non-iterated base structures exhibit relatively inferior and less stable performance.

**Figure 10:**
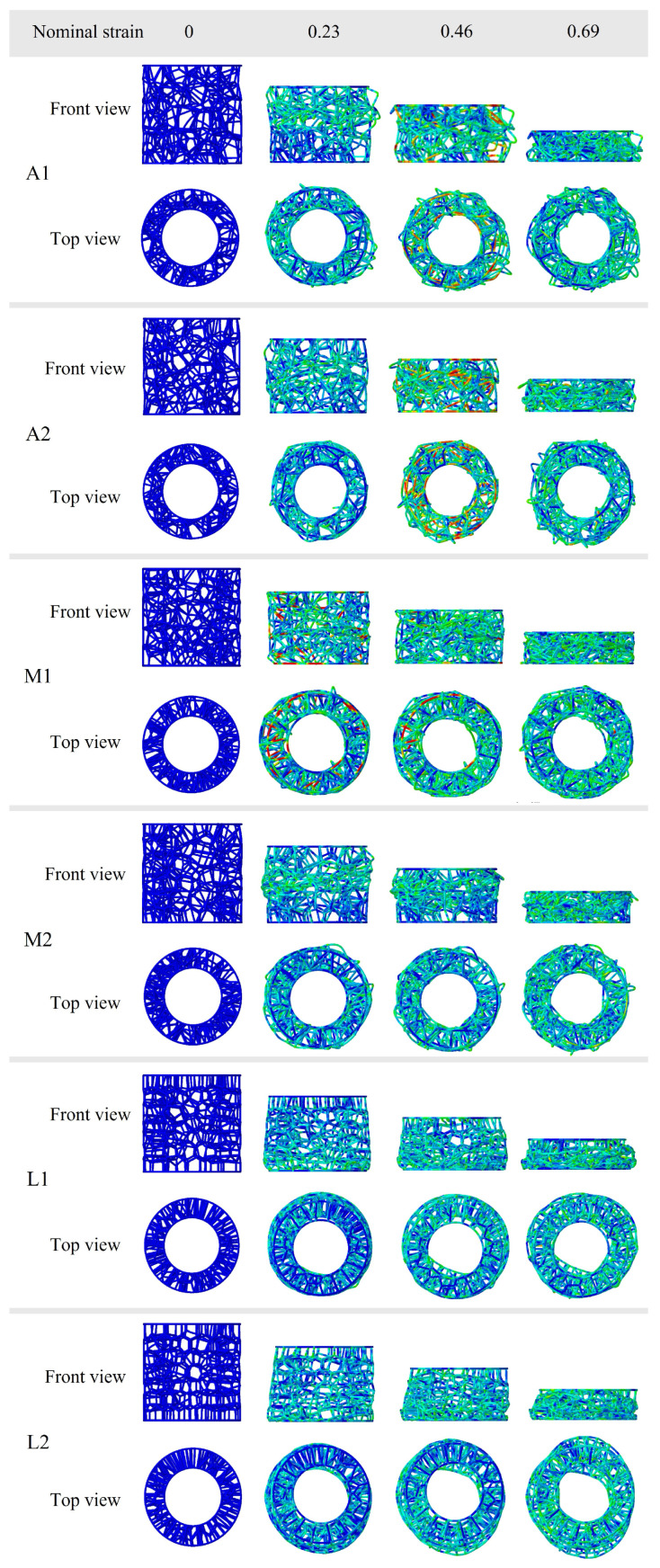
Deformation patterns of the medium-iteration BVT.

Figure 10 illustrates the deformation modes of the BVT structure under axial compression at different iteration times. It can be observed that the high-iteration structures exhibit a unilateral progressive deformation pattern, whereas the base and moderate-iteration structures show varying degrees of middle-layer-first buckling behavior. Specifically, in structure A1, local deformation initiates at an axial strain of 0.23. Upon reaching a strain of 0.46, the structure undergoes multiple local buckling events, accompanied by a secondary peak in the force–displacement curve. This non-uniform deformation pattern is a consequence of the heterogeneous distribution of the internal network, which induces significant fluctuations in energy absorption and precludes the maintenance of a stable, high-performance level. In comparison, structure A2 exhibits enhanced structural homogeneity. This characteristic results in a more uniform deformation mode and a global mechanical response analogous to that of structure M1, leading to close energy absorption performances between them. The moderate-iteration structures exhibit certain optimizations over the baseline type, resulting in improved average performance. Notably, structure M2 displays a pronounced middle-region-dominated buckling pattern, where initial deformation concentrates in the central area and subsequently propagates toward both sides. Such a deformation mode hinders effective internal force transmission, leading to inferior energy absorption compared to A2 and M1. Furthermore, the high-iteration structures L1 and L2 both exhibit a consistent progressive deformation pattern. This is attributed to the increased iterations, which enhance the homogeneity of the internal network and promote the formation of a highly stable deformation mode throughout the structure. It is noteworthy that the mechanical response of the highly iterative BVT structure under compressive loading closely resembles the mechanical properties of the natural luffa sponge, which validates the effectiveness of this bio-inspired design.

## 4. Results and Discussion

The results of the parametric analysis are presented and discussed in this section. The analysis is organized into two parts, focusing on conditions with different mass and equal mass, respectively.

### 4.1. Parametric Analysis Under Different Mass

#### 4.1.1. Influence of *N*

**Figure 11:**
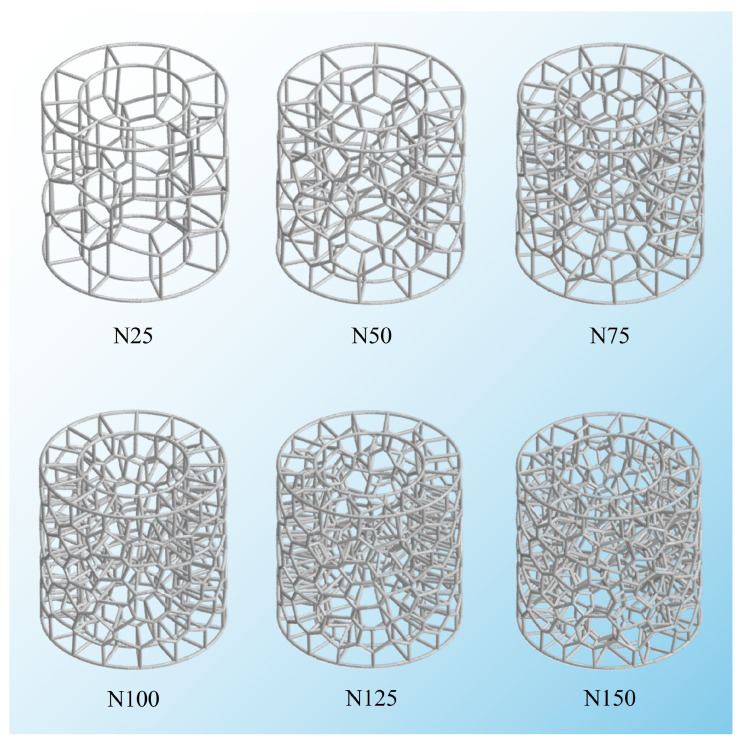
Schematic diagram of BVT structure with different numbers of Voronoi units.

This section examines the influence of *N* of Voronoi units on the energy absorption performance of the BVT structure. Under fixed geometric parameters (*d* = 1 mm, *R*_0_ = 15 mm, *R*_1_ = 25 mm, and *H* = 50 mm), the configurations of BVT structures with different *N* are illustrated in Figure 11 (where the label N25 corresponds to the BVT structure with *N* = 25, and so forth). As can be inferred from the analysis in the previous section, increasing the number of iterations improves the structural performance, which stabilizes after 10 iterations. To eliminate the effect of iteration times, all structures used for comparative study have been optimized over 50 iterations. It is evident that an increase in the *N* leads to a significant rise in the relative density of the BVT structure, and its morphology gradually approaches the porous structure of the natural luffa sponge.

**Figure 12:**
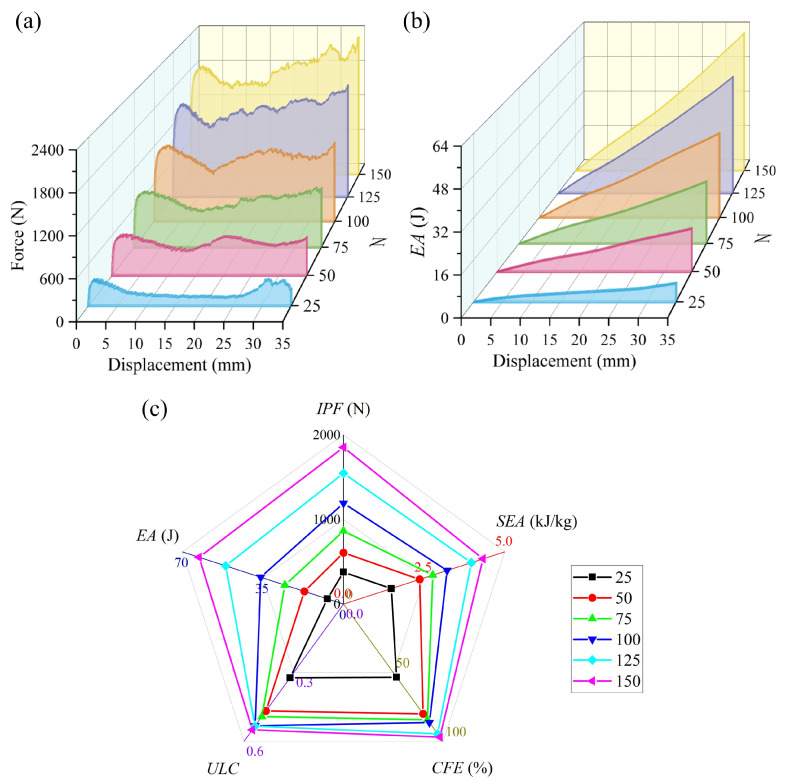
Comparison of energy absorption performance of BVT with different numbers of Voronoi units: (**a**) force–displacement curves, (**b**) energy absorption curves, (**c**) energy absorption performance indicators.

Figure 12 shows a comparison of finite element results for BVT structures under axial compression with six different *N*. As the mesh density increases, all the energy absorption indicators of the BVT structure exhibit significant improvement. Specifically, compared to *N* = 25, when *N* = 150, the *SEA* increases by 191.62%, the *CFE* by 81.61%, the *IPF* by 391.25%, and the *ULC* by 70.56%. During the increase in *N* from 25 to 150, the coupling effect within the BVT structure enhances nonlinearly. Under compressive loading, a denser network facilitates better force transmission, resulting in more uniform stress distribution and more fully developed plastic deformation. The improvement in *SEA* demonstrates that increasing the network density enhances energy absorption performance far more effectively than simply adding mass. Meanwhile, the increase in *ULC* during the densification process can be attributed to the enhanced coupling effect caused by increased contact and interaction among the network members. As density rises, the structure exhibits more frequent fluctuations during compression. It is evident that the overall force–displacement curve approaches the mechanical response of natural luffa sponge under compression. Furthermore, comparing the incremental improvements between adjacent *N* values, the enhancement in overall energy absorption performance is most pronounced when N increases from 25 to 50. In this range, *SEA* increases by 59.20%, *CFE* by 50.00%, and *IPF* by 61.01%. This phenomenon results from the abrupt increase in the spatial density of the BVT network. When N exceeds 50, the force–displacement curve of the BVT structure exhibits a high degree of similarity to the mechanical response of a luffa under compression, characterized by fluctuating plateau behavior following an initial linear elastic stage.

#### 4.1.2. Influence of *d*

In the process of optimization modeling, multiple rod members may converge at a single node in the BVT structure, which requires control over the structural diameter. Therefore, the density of the network affects the adjustable range of the cross-sectional diameter of the rod members. Specifically, as the network density increases, the minimum distance between adjacent members decreases accordingly, leading to a significant reduction in the permissible variation range of the cross-sectional diameter. Hence, to analyze the effect of the cross-sectional diameter *d* on the mechanical properties of the BVT, a network structure with *N* = 125 is established. A comparison between the area of the cross-section and structural mass shows that the two do not exhibit a standard linear correlation. This is because changes in the diameter cause varying degrees of overlapping volume among rod members at the same node.

**Figure 13:**
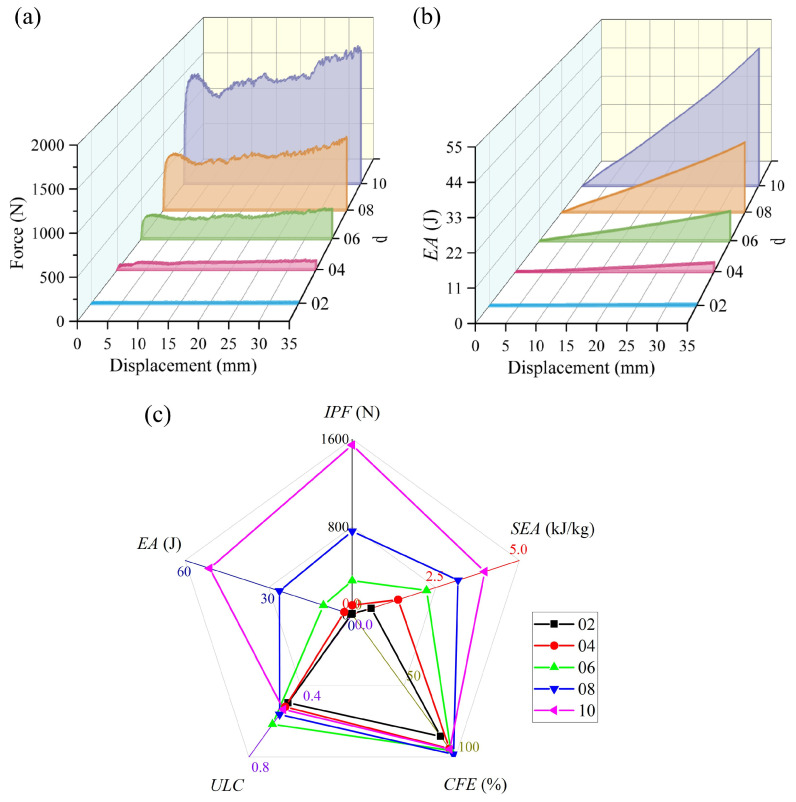
Comparison of energy absorption performance of BVT for different cross-sectional diameters at *N* = 125: (**a**) force–displacement curves, (**b**) energy absorption curves, (**c**) energy absorption performance indicators.

Figure 13 presents the finite element results of the BVT under compressive loading with different *d*. For the BVT structure, an increase in *d* enhances the *SEA*. Meanwhile, a pronounced size saturation effect is observed. Specifically, when the diameter rises from 0.6 mm to 1.0 mm, the *SEA* increases by 78.32%. In contrast, a diameter increase from 2.0 mm to 2.5 mm results in an *SEA* improvement of only 26.36%. This nonlinear response reveals a transition in the energy dissipation mechanism. In sparse network structures, an increase in *d* significantly enhances force transmission within the network, leading to a marked improvement in energy absorption. However, as *d* increases, the network becomes denser. In dense networks, energy dissipation is constrained by redundant contact among members. When *d* exceeds a critical threshold, the densification of the structure shifts the dominant energy dissipation mode from plastic deformation to friction among structural components, ultimately causing the rate of *SEA* improvement to slow down.

### 4.2. Parametric Analysis Under Equal Mass

#### 4.2.1. Synergistic Influence of *N* with *d*

This section discusses the synergistic effects of the *N* with *d* on the energy absorption performance of the BVT structure under a constant mass. Six structures with identical mass are established (maintaining the same geometric parameters *R*_0_ = 15 mm, *R*_1_ = 25 mm, *H* = 50 mm), as shown in Table 1. Among them, label M25 indicates that the structure has *N* = 25, and similarly, six types of structures have partitioned Voronoi cell numbers ranging from 25 to 125. It can be observed that BVT structures with a higher number of partitioned cells correspond to a smaller cross-sectional diameter.

**Figure 14:**
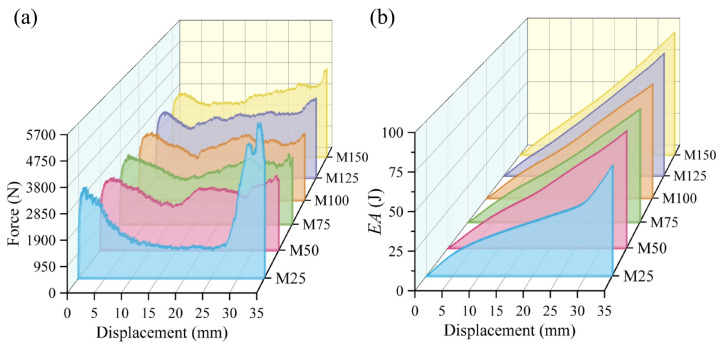
Comparison of the energy absorption performance of BVT under equal mass: (**a**) force–displacement curves, (**b**) energy absorption curves.

**Figure 15:**
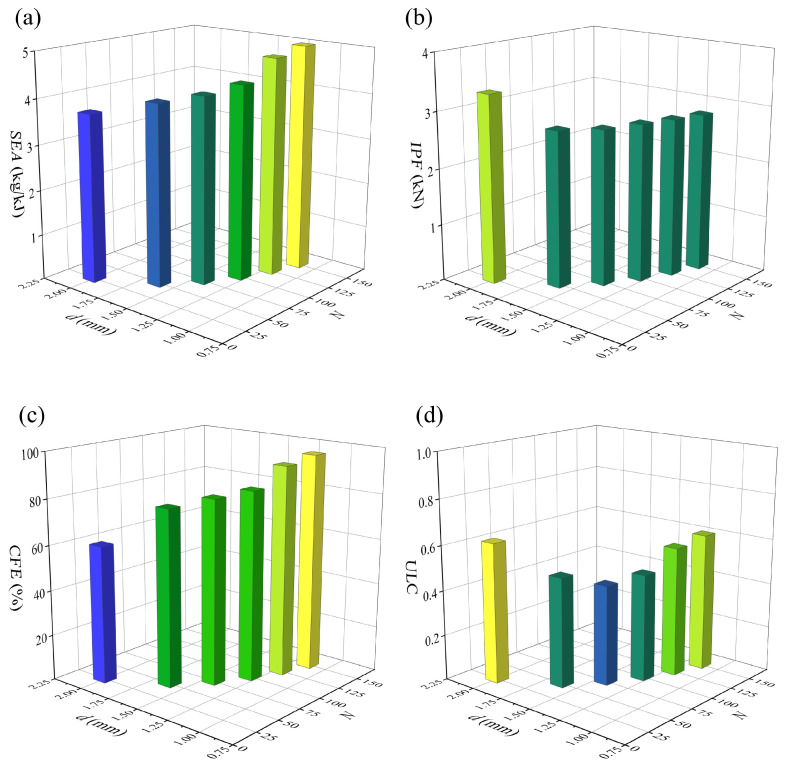
Comparison of energy absorption indicators of BVT under equal mass conditions: (**a**) *SEA*, (**b**) *IPF*, (**c**) *CFE*, (**d**) *ULC*.

The finite element analysis results of six structures under compressive loading are compared in Figure 14 and Figure 15. It is not difficult to observe that, except for M25, the *IPF* values of the other five structures show little variation, with the maximum difference between M75 and M150 being only 2.43%. In contrast, the *IPF* of M25 is significantly higher than that of the other five structures—exceeding that of M150 by 18.09%. Previous single-parameter analyses demonstrate that *N* is positively correlated with the energy absorption performance of the structure, while *d* exhibits an approximately negative correlation with the energy absorption of the BVT structure. However, when both parameters are varied simultaneously, an increase in *N* can offset the adverse effect of reducing *d* on the overall energy absorption. Specifically, both the *CFE* and *SEA* of the structure improve as the number of cells increases and the cross-sectional diameter decreases. Compared with M25, M150 achieves a 34.57% increase in *SEA* and a 58.9% increase in *CFE*. This can be attributed to the fact that a higher *N* results in a denser internal network, which helps to form a more uniform strain field and induces multi-stage buckling modes, thereby dissipating energy more efficiently. On the other hand, a reduction in *d*, while compromising local stiffness, alleviates abrupt fluctuations in load-bearing capacity caused by localized structural rod members. Once *N* exceeds 25, the enhancement in energy absorption attained through structural optimization fully offsets the associated loss in stiffness. This coupled mechanism substantiates that the coordinated optimization of both *N* and *d* constitute an effective strategy for realizing lightweight and high energy-absorption performance in BVT structures.

#### 4.2.2. Influence of *D*_1_

**Figure 16:**
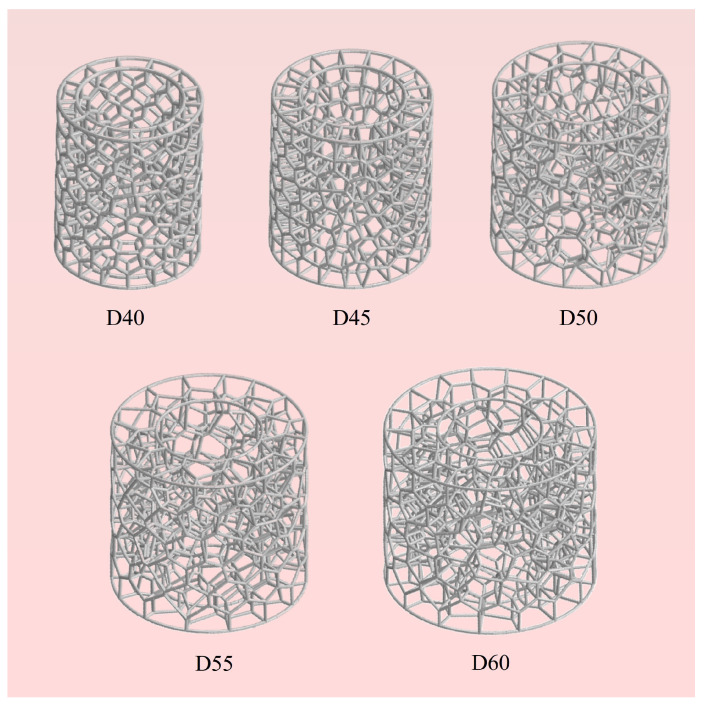
Schematic diagram of BVT structures with different outer diameters with equal mass.

Six BVT structures with identical mass but different outer diameters are constructed, as illustrated in Figure 16. The labeling follows the convention where D40 corresponds to the structure with *D*_1_ = 40 mm. To account for the effect of increased outer diameter on the density of the network structure, *N* in each structure is controlled at 150. Detailed geometric parameters can be found in Table 2.

**Figure 17:**
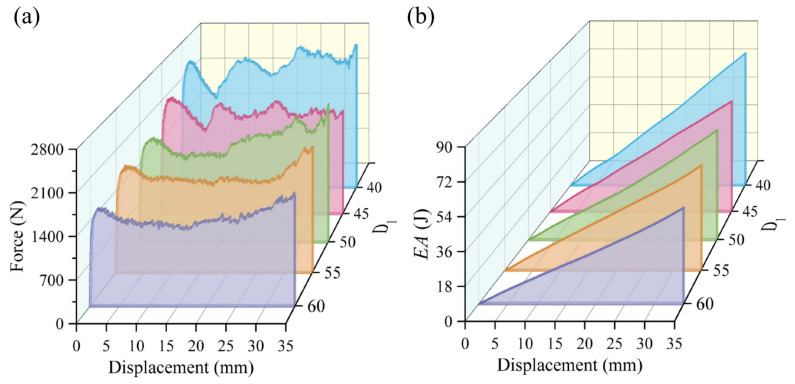
Comparison of energy absorption performance of BVTs with different *R*_1_ under equal mass: (**a**) force–displacement curves, (**b**) energy absorption curves.

**Figure 18:**
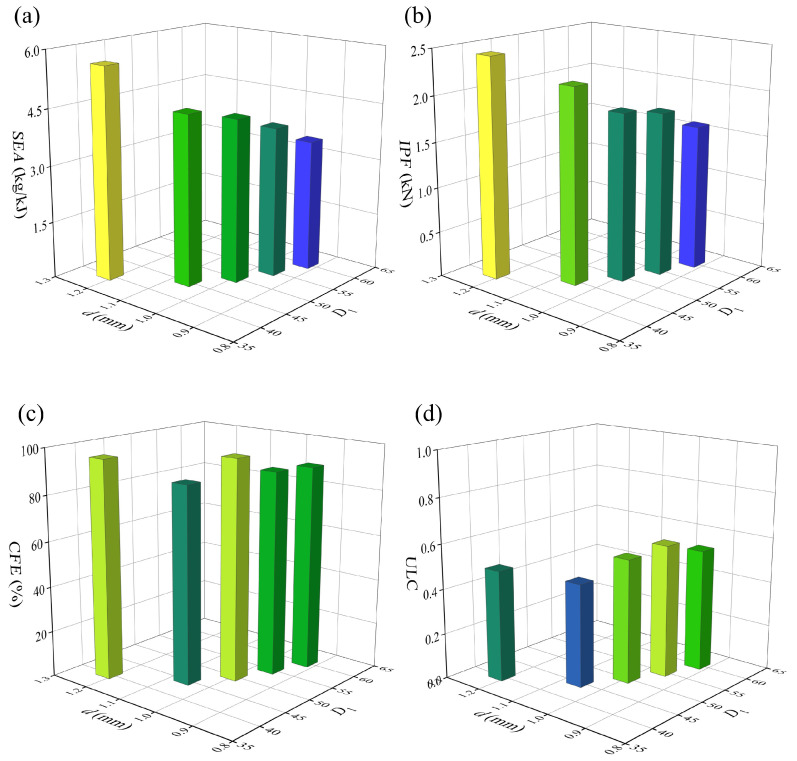
Comparison of energy absorption indicators of BVT with different *D*_1_ under equal mass conditions: (**a**) *SEA*, (**b**) *IPF*, (**c**) *CFE*, (**d**) *ULC*.

Figure 17 and Figure 18 compare the finite element results of five structures under axial compression loading. It can be observed that the energy absorption performance of the BVT structure decreases as its *D*_1_ increases. Specifically, compared with D40, D60 exhibits a 38.18% reduction in *SEA*, a 5.83% decrease in *CFE*, a 34.17% drop in *IPF*, and a 10.30% increase in *ULC*. This performance degradation stems from the fact that, under equal mass and *N*, an increase in *D*_1_ leads to a higher slenderness ratio of the internal rod structure. According to Euler buckling theory, slender members are more susceptible to local buckling instability, which consequently reduces the overall load-bearing capacity. In short, under the condition of constant mass, it is essential to coordinate the relationship between *D*_1_ and rod member slenderness ratio to achieve synergistic optimization of energy absorption performance.

## 5. Comparison with Other Lattices

**Figure 19:**
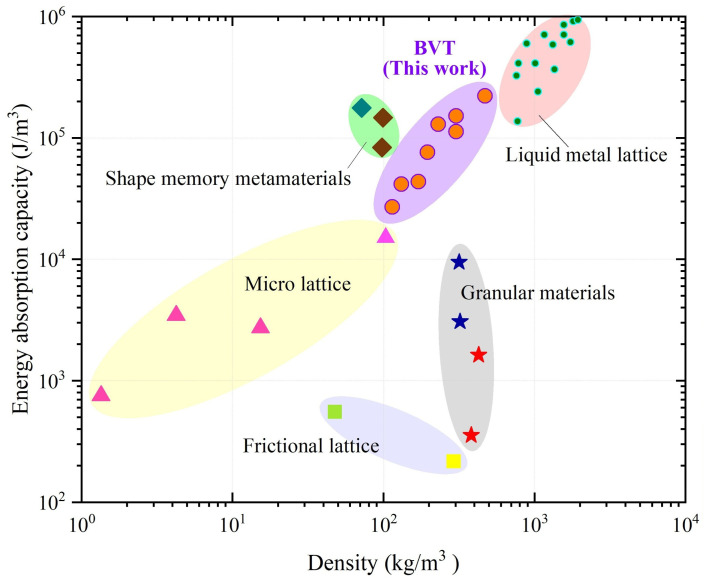
Ashby plot comparing the energy absorption capacity and density of the BVT structure with other typical materials.

The Ashby plot in Figure 19 systematically compares the energy absorption performance and material density of the BVT configuration with those of several conventional material systems: micro lattice [45,46], liquid metal lattice [47], granular materials [48,49], frictional lattice [50,51], and shape memory metamaterials [52,53]. The results show that the present BVT design achieves a more favorable balance between energy absorption and density relative to many established materials.

## 6. Conclusions

Based on the three-dimensional Voronoi diagram method, an innovative structure inspired by the spatial network of luffa sponge is designed and termed the BVT structure. This structure employs randomly distributed three-dimensional Voronoi diagrams to form a disordered network configuration, which surpasses the limitations of conventional ordered cellular structures and demonstrates excellent energy absorption performance. In contrast to traditional approaches that extract design regions from cuboid-based Voronoi partitions, the present work introduces a seed-point density modulation method to generate the network directly within the design domain. The optimized high-density BVT structure demonstrates a more stable stress–strain plateau region during compression, and its mechanical behavior closely resembles that of natural luffa sponge structures. The main conclusions are as follows:

(1) After 50 optimization iterations, the structural network connectivity and distribution uniformity are significantly enhanced, with the deformation mode exhibiting an ideal progressive evolution and the energy absorption efficiency achieving notable enhancement.
(2) As *N* increases, the density of the BVT structure correspondingly rises, resulting in a significant enhancement of energy absorption performance that approaches both the morphology and energy absorption level of natural luffa sponge.
(3) Within a certain range, increasing the *d* of the members enhances structural stiffness and simultaneously improves energy absorption. For instance, a rise in *d* from 0.6 mm to 1.0 mm results in a 78.32% increase in the *SEA*.
(4) Under equal mass, a distinct synergy exists between the *N* and *d* of the BVT. “Looseness” configurations—higher *N* with smaller *d*—demonstrate superior *SEA*. For instance, M150 exhibits a 34.57% higher *SEA* than M25. However, this trend is not universal. Under constant mass, “looseness” achieved by coordinating the *d* and *D*_1_ can reduce *SEA*.

## Figures and Tables

**Table 1:** Geometric parameters and mass of BVT with equal mass.

Structure Number	*d* (mm)	*N*	*m* (g)
M25	1.000	25	19.0
M50	0.812	50	19.0
M75	0.718	75	19.0
M100	0.652	100	19.0
M125	0.607	125	19.0
M150	0.572	150	19.0

**Table 2:** Geometric parameters and mass of BVTs with different *D*_1_ with equal mass.

Structure Number	*d* (mm)	*D*_1_ (mm)	*m* (g)
D40	1.215	40	14.5
D45	1.061	45	14.5
D50	1.000	50	14.5
D55	0.963	55	14.5
D60	0.930	60	14.5

## Data Availability

The original contributions presented in this study are included in the article. Further inquiries can be directed to the corresponding author.
